# Gender difference in early initiation of methamphetamine use among current methamphetamine users in Muse, Northern Shan State, Myanmar

**DOI:** 10.1186/s12954-017-0147-0

**Published:** 2017-05-08

**Authors:** Yu Mon Saw, Thu Nandar Saw, Junko Yasuoka, Nyein Chan, Nang Pann Ei Kham, Wint Khine, Su Myat Cho, Masamine Jimba

**Affiliations:** 1 Department of Healthcare Administration, Graduate School of Medicine, Nagoya University, 65 Tsurumai-cho, Showa-ku, Nagoya, 466-8550 Japan; 2 Nagoya University Asian Satellite Campuses Institute, Nagoya, Japan; 3 Myanma Perfect Research, Yangon, Myanmar; 4 Department of Community and Global Health, Graduate School of Medicine, University of Tokyo, Tokyo, Japan; 5 Department of Social Research, Defence Services Medical Research Centre, Tatkone Township, Nay Pyi Taw, Myanmar; 6 Institute for Drug and Alcohol Studies, Virginia Commonwealth University, Richmond, VA USA; 7 Save the Children, Bahan Township, Yangon, Myanmar

**Keywords:** Initiation, Methamphetamine, Gender, Drug user, Myanmar

## Abstract

**Background:**

Globally, methamphetamine (MA) use is a significant public health concern due to unprecedented health effects of its use. However, gender similarities and differences in early age of MA initiation and its risk factors among current MA users have been understudied in a developing country setting.

**Methods:**

A community-based, cross-sectional study was conducted using a computer assisted self-interviewing program from January to March 2013 in Muse, Northern Shan State, Myanmar. A total of 1362 (775 male and 587 female) self-reported current MA users aged between 18 and 35 years were recruited using respondent-driven sampling. Two gender-stratified multiple logistic regression models (models I and II) were done for analysis.

**Results:**

For similarities, 73.0% of males and 60.5% of females initiated MA before their 18th birthday. The early age of MA initiation was positively associated with the reasons and places of the first time MA use among both genders. For differences, males [hazard ratio 1.35; 95% confidence interval, 1.18–1.54] had a significantly higher risk than females to initiate MA at earlier age. Among male users, participants who had bisexual/homosexual preferences were more likely to initiate MA use earlier. In contrast, female users who exchanged sex for money and/or drugs were more likely to initiate MA in earlier age.

**Conclusions:**

More than 60.0% of male and female participants initiated MA use early; however, males initiated use earlier than females. Although similarities were found among both genders, differences found in key risk factors for early age MA initiation suggest that gender-specific, MA prevention programs are urgently needed in Myanmar.

## Background

Amphetamine-type stimulants (ATS) are getting popular among young population, due to their affordable and wide availability [1, 2]. In 2012, ATS was ranked as the second most commonly used drug worldwide which may be due to its powerful stimulant effects. It acts in the central nervous system: increasing alertness; providing a sense of increased energy, concentration, physical strength; and creating euphoria [3]. Among different types of ATS, methamphetamine (MA) pills are most commonly used all over the world. Approximately 80 million people used ATS, and 21 million of them were based in East and Southeast Asia [1, 4, 5].

The response to MA is not always the same between males and females. Gender-specific similarities and variations have been reported in many aspects of MA use and health services [6]. For example, both males and females may use MA for sexual enhancement [7], but females are more likely to use MA pills for weight loss [8, 9]. Females also tend to show more dependence and commitment to MA, whereas males are more likely to use other drugs if MA is not available [10, 11]. Females seek access to MA abuse treatment more often than males, [2] and tend to be more open and responsive to this treatment [12]. Moreover, being compared to male users, female users seemed to have a tendency to initiate MA, cocaine, and amphetamine at earlier ages, and to report medical problems caused by drug abuse than male users [11, 13].

Initiation of MA occurs sometime during adolescence. Initiation at such an early age is often associated with a variety of negative outcomes in adulthood including drug addiction, criminal, and violent behavior, and health-related problems [14, 15]. In addition, individuals who start using drug in their adolescence may continue using it through their adulthood. Even when they stop, they might revert into using it later in adulthood [16, 17]. Thus delaying the initiation of MA use, in addition to preventing MA use, could be important in controlling the adverse effects of MA use.

In Myanmar, the government has tried to control opiate drug use, but has shown less attention to ATS. This has led to Myanmar becoming a major ATS manufacturing country in Southeast Asia; in particular, it has produced MA pills known as “Yama or Yaba” [1, 4]. These drugs are mainly produced for the international market, but MA is also used within the country. In 2009, MA pills were seized in 16 out of 17 administrative regions in Myanmar, highlighting their nationwide availability [18]. Although, opium and heroin remain the most widely used drugs in Myanmar [18].

Despite increasing MA use, little has been studied about the initiation of MA, particularly the gender similarities and differences in it, in Myanmar. Delaying the initiation is critical in this country, but the risk factors for initiation have been adequately studied. It is important to know the age at which MA use is initiated and factors associated with this in Myanmar. Such information could help establish effective strategy for controlling MA use in the country. Moreover, evidence is particularly lacking in relation to gender-specific factors associated with the age of initiation among drug users in Myanmar. Therefore, this study was conducted to elucidate the gender-specific factors associated with the early initiation of MA use.

## Methods

### Study settings and participants

The study area was Muse city in Northern Shan State, Myanmar. The city is located close to the Chinese border, neighboring Ruili city in the Chinese province of Yunnan, and accommodates a highly mobile population of migrant workers. Northern Shan State has a long history of opium and heroin use [19], and MA use has also become a growing problem in this area over the past decade. The majority of MA pill seizures in Myanmar are routinely made near to the production areas in the border towns of Northern Shan State.

A community-based, cross-sectional survey was carried out from January to March 2013. In total, 1385 MA users (782 males and 603 females) were recruited. Of them, 23 participants (7 males and 16 females) were excluded from final data analysis due to withdrawal from the study or who missed to answer the outcome question. A final sample of 1362 MA users (775 males; 587 females) were considered for analysis. The study inclusion criteria were: (1) self-reported MA users aged between 18 and 35 years, (2) those who had used methamphetamine drugs in the last 3 months, (3) those who had no withdrawal symptoms and were not under the influence of drugs at the time of interview, (4) those who are able to read the Myanmar language, and (5) those who voluntarily participated in the study by giving an informed consent.

A computer assisted self-interviewing (CASI) method was applied, in which participants read and respond to survey questions on a computer screen. Since CASI is a self-administered computer program, it improves data collection concerning socially undesirable behaviors [20–22]. Each CASI section took 35–45 min to complete. Various private settings were used for data collection, based on the participants’ preference, such as in a motel room, at the participant’s house, at their place of drug use, at their place of work, or in the project’s car. For each interview, two or more research team members visited the interview place and set up computer for CASI.

### Data collection procedure and recruitment protocol

The study participants were recruited by respondent-driven sampling (RDS) [23]. First, an MA user was recruited from a local drug user network as the first study participant from the first initial recruitment arm. In total, eight initial recruitment arms were recruited with the help of the National Drug User Network, local men who had sex with men (MSM) network, brothels, local highway car associations, and local youth volunteer groups. The three initial recruitment arms stopped either at second or third wave out of eight initial recruitment arms (4 males and 4 females) [24]. In total, 4095 coupons were distributed and 1427 coupons were returned. Among returned coupons, 62 coupons that presented after two-week coupon expiration date were excluded from the study.

The same recruitment protocol was used to recruit male and female MA users, and they turned to recruit both genders. They also recruited MA users from different settings of the population including MSM, FSWs, students, laborers, housewives, and highway drivers. The already recruited MA users recruited three other MA user friends after completing their interviews. Each respondent was allowed to recruit up to three other people to participate in the survey within the two-week coupon expiration date.

All the participants received 2000 Kyats (approx. US$ 2.5) as an incentive directly following their interviews. They were also eligible to receive a secondary incentive: a steel cup (for the students, laborers, housewives, and highway drivers) or lubricant gel (for FSWs and MSM) that was equivalent to 900 Kyats (approx. US$ 1.2) if their recruited participants completed their interviews [24]. However, MA users also allowed to choose a steel cup or lubricant gel depending on their preference as a secondary incentive. The other respondents were given the same opportunities as the first respondents for further recruitment and incentives.

### Measures

The questionnaire was adapted from ones created by the United Nations Office on Drugs and Crime [25]. It was translated from English to the Myanmar language and was pre-tested among a sample of MA users in October 2012. Back-translation was done from Myanmar language to English before and after the pre-test to ensure semantic equivalence. A questionnaire was modified based on the results of the pre-test to make it more understandable and easier for participants to answer. Three Myanmar public health experts, who are working on drug use and sexual behavior issues in Myanmar, also reviewed the study questionnaire.

An MA user was defined as such if he/she had used MA at least three times in the 90 days prior to the interview [26]. The outcome variable was the age at which MA use began, which was measured as a binary variable (early versus late), based on the average age of high school completion, which was 17 years old. At this age, most of the students become independent, as they start working or go to college/university [27]. Independent living may lead to poor parental supervision and monitoring which may be responsible for the increased risk of the initiation of substance use [28, 29]. The early age of initiation for MA use was set up as 17 years or younger; then it was dichotomized as “1” and “0” otherwise. Other covariates included ethnicity, behaviors related to MA use (i.e., reason for first-time MA use, sources of access to MA during first use, and route of administration during first use), sexual behaviors [having ever exchanged sex for money or drugs, been diagnosed with a sexually transmitted infection (STI), and had multiple sexual partners within the past 6 months], having ever had suicidal thoughts, and instances of previous suicide attempts.

### Statistical analysis

The Statistical Package for the Social Sciences (SPSS) 18.0 software (Chicago, Illinois, USA) was used for statistical analyses. First, a descriptive analysis was run for socio-demographic characteristics, sexual risk behaviors, and characteristics related to MA use by gender. The Kaplan-Meier curve was plotted, and the log-rank test performed to compare gender differences regarding early initiation of MA use. To determine factors associated with early age of MA initiation, two gender-stratified multiple logistic regression models (models I and II) were performed. In all the analyses, the level of significance was set at *p* < 0.05 (two-tailed).

### Ethical considerations

The study protocol and consent procedure were approved by the Research Ethics Committee of the Graduate School of Medicine, the University of Tokyo, Tokyo, Japan (Ref. no: 10006/2012), and the Ethical Review Committee, the Defence Services Medical Research Centre, Nay Pyi Taw, Myanmar (Document No: 1/2/IRB-7/2013). The purpose of the study and CASI interview procedures were clearly explained to each participant prior to the interview by research team members. For individual interviews, the privacy needs and personal preferences of each respondent were given special attention. All the participants were given time to decide on their voluntary participation and involvement. They were also informed that they could skip answering any question that they did not want to answer and could withdraw from participation at any time during or after the interview without penalty. If participants fully understood and decided to participate, they were requested to read about informed consent on the computer screen and click “agree to participate in this survey” to answer the survey questions. A computer-based informed consent form mentioning the study objectives and purpose of the research was obtained from all respondents, and the confidentiality of the entire data were carefully maintained.

## Results

### Socio-demographic characteristics of participants

The socio-demographic characteristics of all the participants are shown in Table 1. Of 1362 participants, 56.9% (*n* = 775) were males, and their mean age was 23.4 years [standard deviation (SD) 3.6], and the mean age of females was 22.7 (SD 3.4). More than half of males (54.3%) and females (54.2%) were aged between 21–25 years. Among 1362, 936 (68.7%) reported as being single, with the remainder reporting as either currently married (426, 31.3%) or ever married. Regarding educational status, 607 (44.6%) participants reported having a secondary level of education, while 302 (22.2%) reported having university education (*p* < 0.001). In total, 983 (72.2%) participants were employed at the time of the interview (*p* = 0.001).

**Table 1:** Socio-demographic characteristics of participants (*N* = 1,362)

Characteristics	Total (*N* = 1,362)		Male (*n* = 775)		Female (*n* = 587)		*P* value
	*n*	%	*n*	%	*n*	%	
Age^a^ (year)							
≤20	336	24.7	165	21.3	171	29.1	<0.001
21–25	739	54.3	421	54.3	318	54.2	
26–30	223	16.3	145	18.7	78	13.3	
>30	64	4.7	44	5.7	20	3.4	
Marital status							
Never married	936	68.7	553	71.4	383	65.2	0.016
Ever married	426	31.3	222	28.6	204	34.8	
Education							
Primary school	132	9.8	54	7.0	80	13.6	<0.001
Secondary school	607	44.6	304	39.2	303	51.6	
High school	319	23.4	210	27.1	109	18.6	
University	302	22.2	207	26.7	95	16.2	
Ethnicity							
Shan	461	30.6	213	27.5	203	34.6	<0.001
Kachin	274	20.1	158	20.4	116	19.8	
Burma	409	30.0	218	28.1	191	32.5	
Others^*^	263	19.3	186	24.0	77	13.1	
Employment status							
Unemployed	379	27.8	188	24.3	191	32.5	0.001
Employed	983	72.2	587	75.7	396	67.5	

^a^Mean age 23.4 years (standard deviation, SD 3.46) for males and 22.7 years (SD 3.38) for females; *(Kyar, Kayin, Chin, Mon, Rakhin, Multi-ethnic, Indians, and the Chinese);

### Gender differences in age of methamphetamine use

**Fig. 1:**
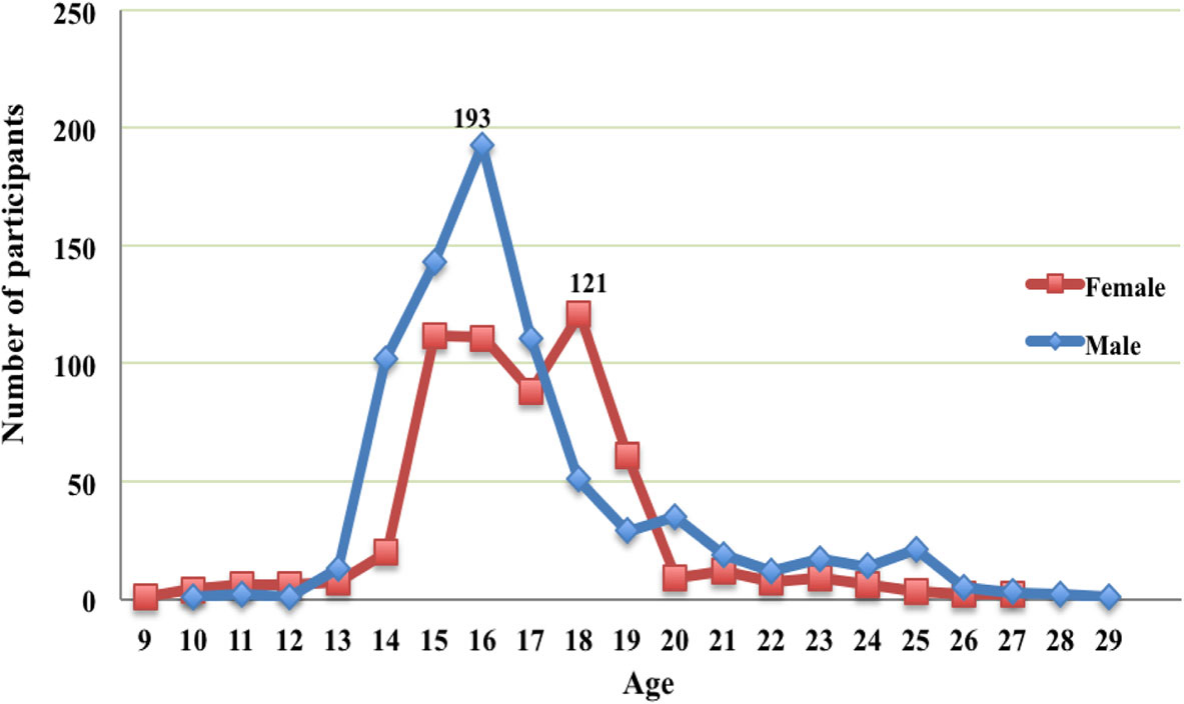
Age at initiation of methamphetamine use, stratified by gender

Figure 1 shows the percentage of ages at initiation of MA use, stratified by gender. A rapid increase in initiation occurred at 14 years for males and 15 years for females. The peak age of initiation was 16 years for males (*n* = 193, 24.9%) and 18 years for females (*n* = 121, 20.6%). The median age at initiation of MA use was 16 years (age range: 10–29) for males and 17 years (age range: 9–27) for females.

**Fig. 2:**
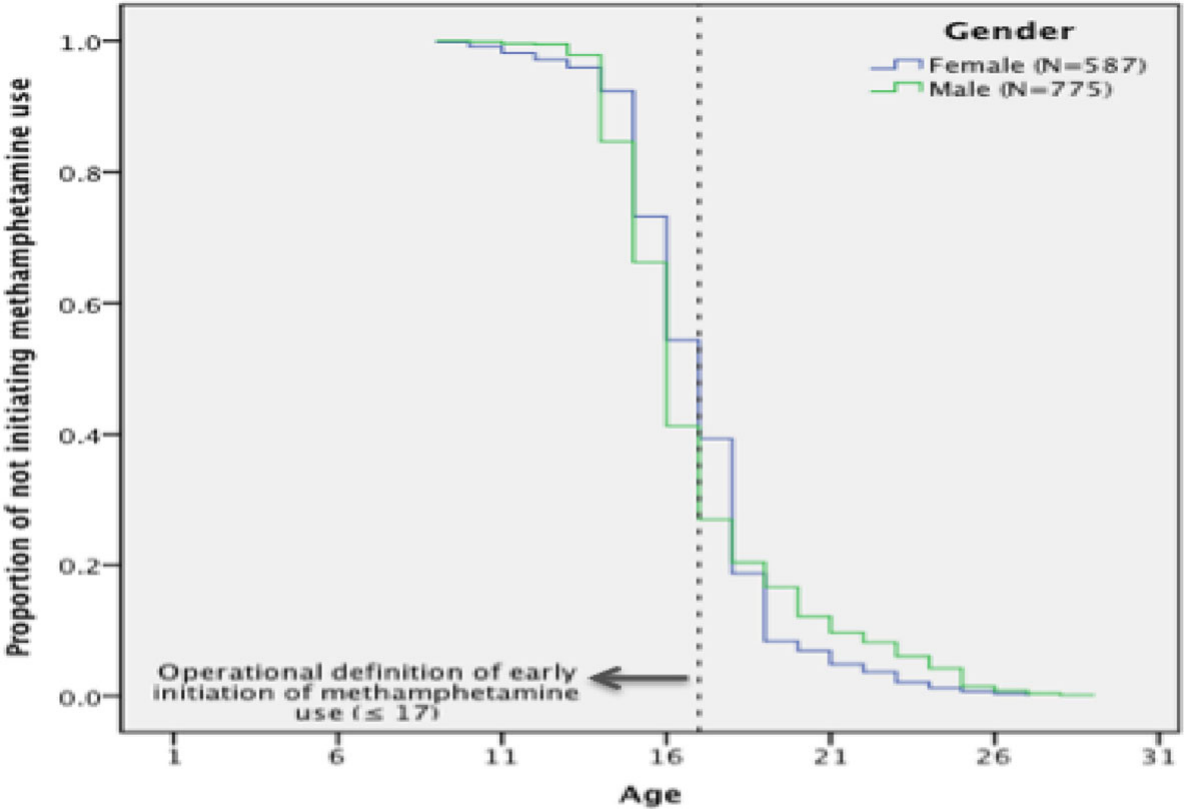
The Kaplan-Meier curve depicting age at initiation of methamphetamine use by gender

Figure 2 presents the Kaplan-Meier estimates of age at initiation of MA use by gender. Male users showed a significantly higher risk of early initiation [hazard ratio 1.35; 95% confidence interval, 1.18–1.54] than did female users.

### Multiple logistic regression analyses of early initiation of methamphetamine use, stratified by gender

Tables 2 and 3 show the results of the multiple logistic regression analyses for males and females. In model I, socio-demographic factors (i.e., age, marital status, education, ethnicity, employment status, and sexual orientation) and characteristics associated with first-time MA use (i.e., place of first MA use, reason for first MA use, source of first-time access to MA, and route of administration during first-time use) were included. In model II, risky sexual behaviors (i.e., having ever exchanged sex for money and/or drugs and having ever been diagnosed with an STI), and suicidal behaviors were added to the variables in model I.

**Table 2:** Multiple logistic regression analysis of factors associated with early initiation of methamphetamine use among males (*n* = 775)

			Model I^a^		Model II^b^	
	*n*	%	AOR	95% CI	AOR	95% CI
Age (current age at survey)						
≤23	437	56.4				
>23	338	43.6	0.39	0.25–0.60	0.38	0.24–0.60
Marital status						
Never married	553	71.4				
Ever married	222	28.6	0.78	0.51–1.17	0.74	0.49–1.13
Education						
Secondary school and higher	358	46.2				
High school and higher	417	53.8	0.71	0.48–1.05	0.73	0.49–1.08
Ethnicity						
Shan	213	27.5				
Kachin	158	20.4	0.62	0.35–1.11	0.58	0.32–1.05
Burma	218	28.1	0.61	0.36–1.05	0.56	0.32–0.97
Others*	186	24.0	0.37	0.22–0.63	0.35	0.20–0.61
Employment status						
Unemployed	188	24.3				
Employed	587	75.7	0.72	0.43–1.20	0.69	0.40–1.19
Sexual orientation						
Heterosexual	325	41.9				
Bisexual/homosexual	450	58.1	1.57	1.07–2.29	1.58	1.06–2.33
Place of first methamphetamine use						
House (own/drug dealer’s/ friend’s/sexual partner’s)	218	28.1				
School/dormitory/workplace	121	15.6	1.57	0.91–2.71	1.55	0.89–2.71
Entertainment venues^#^	436	56.3	3.08	2.03–4.65	3.00	1.97–4.58
Reason for first methamphetamine use						
Encouraged by friend/sexual partner/drug dealers	308	39.7				
For weight loss/Work-related purposes	173	22.3	1.61	1.02–2.54	1.54	0.97–2.45
Curious about methamphetamine effects/for fun	294	38.0	2.84	1.74–4.65	2.60	1.58–4.28
Source of first-time access to methamphetamine						
Sexual exchanged/freely offered/provided by employer	353	45.5				
Got someone to buy/bought oneself	422	54.5	2.57	1.77–3.71	2.49	1.71–3.62
Route of administration of first time use						
Inhalation	727	93.8				
Drank/smoked/injected	48	6.2	1.18	0.56–2.48	1.13	0.53–2.42
Ever had exchanged sex for money and/or drugs						
No	241	31.1				
Yes	534	68.9			0.98	0.65–1.49
Ever diagnosed with an STI						
No	345	44.5				
Yes	430	55.5			1.15	0.77–1.72
Ever experienced suicidal ideation						
No	376	48.5				
Yes	399	51.5			0.82	0.55–1.22
Ever attempted suicide						
No	640	82.6				
Yes	135	17.4			2.27	1.27–4.07

*OR* odd ratio, *CI* confidence interval, *AOR* adjusted odd ratio; *(Kyar, Kayin, Chin, Mon, Rakhin, Multi-ethnic, Indians, and the Chinese); ^#^(club/karaoke/disco/restaurant/hotel/guest house/game center)

^a^Model I adjusted for age, marital status, education, ethnicity, employment status, sexual orientation, place of first methamphetamine use, reason for first methamphetamine use, source of first methamphetamine access, and route of administration during first-time use

^b^Model II adjusted for age, marital status, education, ethnicity, employment status, sexual orientation, place of first methamphetamine use, reason for first methamphetamine use, source of first access to methamphetamine, route of administration during first-time use, ever exchanged sex for money and/or drugs, ever been diagnosed with an STI, ever experienced suicidal ideation, and ever attempted suicide

**Table 3:** Multiple logistic regression analysis of factors associated with early initiation of methamphetamine use among female participants (*n* = 587)

			Model I^a^		Model II^b^	
	*n*	%	AOR	95% CI	AOR	95% CI
Age (age during the survey period)						
≤23	358	61.0				
>23	229	39.0	0.85	0.52–1.39	0.85	0.51–1.41
Marital status						
Never married	383	65.2				
Ever married	204	34.8	0.97	0.63–1.48	0.96	0.62–1.49
Education						
Secondary school and lower	383	65.2				
High school or higher	204	34.8	1.40	0.93–2.08	1.30	0.85–1.99
Ethnicity						
Shan	203	34.6				
Kachin	116	19.8	0.51	0.31–0.85	0.48	0.29–0.81
Burma	191	32.5	0.60	0.35–1.00	0.61	0.36–1.04
Others^*^	77	13.1	0.63	0.34–1.16	0.62	0.33–1.16
Employment status						
Unemployed	191	32.5				
Employed	396	67.5	0.71	0.47–1.07	0.65	0.42–1.02
Place of first methamphetamine use						
House (own/drug dealer’s/friend’s/sexual partner’s)	373	63.5				
School/dormitory/workplace	39	6.7	2.56	1.19–5.53	2.82	1.28–6.19
Entertainment venues^#^	175	29.8	4.06	2.67–6.17	4.24	2.77–6.50
Reason for first methamphetamine use						
Encouraged by friend/sexual partner/drug dealers	242	41.2				
For weight loss/ work-related purposes	190	32.4	1.89	1.21–2.93	1.61	1.02–2.53
Curious about methamphetamine effects/for fun	155	26.4	3.30	2.00–5.45	3.16	1.89–5.27
Source of first-time access to methamphetamine						
Sexual exchange/freely offered/provided by employer	354	60.3				
Got someone to buy/bought oneself	233	39.7	1.09	0.74–1.59	1.05	0.72–1.56
Route of administration of first time use						
Inhalation	580	98.8				
Drank/smoked/injected	7	1.2				
Ever had exchanged sex for money and/or drugs						
No	211	35.9				
Yes	376	64.1			2.04	1.28–3.26
Ever diagnosed with an STI						
No	308	52.5				
Yes	279	47.5			1.21	0.76–1.92
Ever experienced suicidal ideation						
No	243	41.4				
Yes	344	58.6			1.10	0.72–1.68
Ever attempted suicide						
No	450	76.7				
Yes	137	23.3			0.98	0.60–1.60

*OR* odd ratio, *CI* confidence interval, *AOR* adjusted odd ratio; *(Kyar, Kayin, Chin, Mon, Rakhin, Multi-ethnic, Indians, and the Chinese); ^#^ (club/karaoke/restaurant/hotel/guest house/game center)

^a^Model I adjusted for age, marital status, education, ethnicity, employment status, place of first methamphetamine use, reason for first methamphetamine use, and source of first-time access to methamphetamine

^b^Model II adjusted for age, marital status, education, ethnicity, employment status, place of first methamphetamine use, reason for first methamphetamine use, source of first-time access to methamphetamine, ever exchanged sex for money and/or drugs, ever diagnosed with an STI, ever experienced suicidal ideation, and ever attempted suicide

Among males, in model I, factors associated with an increased likelihood of early initiation of MA use included having bisexual/homosexual preferences (adjusted odds ratio [AOR] = 1.57, 95% CI: 1.07–2.29), having used MA at entertainment venues (AOR = 3.08; 95% CI: 2.03–4.65), having used MA either for weight loss or work-related purposes (AOR = 1.61; 95% CI: 1.02–2.54), having used MA for the first time because of curiosity about its effects or for fun (AOR = 2.84; 95% CI: 1.74–4.65), and having bought MA by oneself or gotten someone else to buy it (OR = 2.57; 95% CI: 1.77–3.71). Conversely, older age (AOR = 0.39; 95% CI: 0.25–0.60) and being employed (AOR = 0.72; 95% CI: 0.43–1.20) were negatively associated with early initiation of MA use. In model II, the socio-demographic factors and characteristics associated with first-time MA use were statistically significant, and suicide attempts were positively associated with early initiation (AOR = 2.27; 95% CI: 1.27–4.07) (Table 2).

For females, in model I, multiple logistic regression analysis revealed that participants belonging to the Kachin (AOR = 0.51; 95% CI: 0.31–0.85) ethnic groups were less likely to report early initiation of MA use. In contrast, participants who used MA for the first time at an entertainment venue (AOR = 4.06; 95% CI: 2.67–6.17) and at a school, dormitory, or the workplace (AOR = 2.56; 95% CI: 1.19–5.53) were more likely to report early initiation of MA use. Similar associations were found among those whose reasons for first-time MA use was either weight loss or work-related purposes (AOR = 1.89; 95% CI: 1.21–2.93) and curiosity about the drug’s effects or for fun (AOR = 3.30; 95% CI: 2.00–5.45). In model II, the characteristics associated with first-time MA use were statistically significant and participants who had ever exchanged sex for money and/or drugs (AOR = 2.04; 95% CI: 1.28–3.26) were more likely to have initiated MA use earlier (Table 3).

## Discussion

In this study, gender similarities and differences were found with regard to both the early initiation of MA use and the associated risk factors. With regard to similarities, a high rate of early initiation was observed in both genders. The places and reasons for first-time MA use were positively associated with early initiation of MA use among both males and females. Participants of both genders within the Kachin ethnic group were less likely to initiate MA use at an early age, as compared to those of the Shan ethnic group. With regard to differences, males tended to initiate MA use earlier than females. Among male MA users, those who had bisexual/homosexual preferences had a higher likelihood of reporting early initiation of MA use. Furthermore, male MA users who were employed at the time of the survey were less likely to report early initiation of MA use. Among female MA users, exchanging sex for money and/or drugs were positively associated with early initiation.

One of the marked similarities between the genders was the high percentage of MA use within the sample. More than half of males (73.0%) and females (60.5%) initiated MA use before the age of 18 years. Such a high proportion of early initiation of MA use is a major concern, when considering its potential association with a range of health problems later in life [14, 15].

Both males and females belonging to the Kachin ethnic group were less likely to report early initiation of MA use, as compared to participants belonging to the Shan ethnic group. In the United States of America, the age at which use of MA is initiated has also been associated with ethnicity [30]. Similar to MA, early initiation of smoking and the use of alcohol and illicit drugs varied across racial and ethnic groups [31–34].

The place of MA use was also similar across genders. Notably, using MA for the first time at entertainment venues was found to be a potential risk factor for early initiation. This is because entertainment venues, such as bars, clubs, restaurants, motels, Karaoke bars, and game centers provide good opportunities for dealers to sell drugs; drug users can also easily access MA and other illicit drugs at such places [35, 36]. Furthermore, MA is regarded as a “club or party drug” in the West [36–39]; a similar trend has been observed in Asia. Visiting an entertainment venue is also often associated with drinking and smoking, and the consumption of other illegal substances, such as cocaine and marijuana [40, 41]. In the same way, entertainment venues were found to be one of the important factors in the early use of MA.

Several differences were found between males and females. First, males were more likely to initiate MA use earlier than females were. This is consistent with previous studies conducted in the United States of America [42, 43], but not with a study in Taiwan [44] and another study conducted United States of America [45]. Males tend to initiate MA use earlier, as female users are more likely to be stigmatized by society than male users [46]. Drug use is a violation of social norms of behavior; thus, many people consider drug use by females as even worse than by males [47, 48]. This could also hold true for our study population. In addition, MA use may be influenced by the working environment. Since our study area is close to a Chinese border city, males usually take up jobs during early adolescence, thereby potentially gaining more exposure to MA through their workplaces. One study conducted in the United States of America also indicated that males are more likely than females to come across opportunities to use marijuana, cocaine, hallucinogens, and heroin [49].

Although MA initiation was associated with work purposes for both males and females, differences between males and females in the reasons provided for MA use were worth noticing. For example, females tended to initiate MA use to help them lose body weight, while males initiated it for fun or curiosity about the effects thereof; these results are similar to those of previous studies [11, 50–52]. Indeed, MA users sometimes lose weight dramatically when using the drug for a substantial period, as it causes loss of appetite [42, 53, 54]. However, rapid weight gain may occur following the cessation of MA. This makes it more difficult for the affected MA users to stop, resulting in the development of dependence on the drug [55].

Gender differences were also found for behaviors posing an HIV risk to the participants. For example, among female MA users, exchanging sex for money and/or drugs was associated with early initiation of MA use. The exchange of sex for money and/or drugs may be a means for their survival. This is supported by the fact that other factors that are associated with initiation of drug use are homelessness or running away from home [56, 57]. It can also be a means to quell MA addiction [56, 57]. Associations between MA initiation and risky sexual behaviors have an important implication for the fight against HIV/AIDS and other STIs. Thus, it is important to understand the factors associated with high-risk sexual behaviors.

This study provides several important findings and insights. However, some limitations should be noted. First, there is a possibility of recall bias in the reported ages of first MA use, although people may remember this kind of moment better than more ordinary occasions [58]. Second, the possibility of underreporting exists, particularly for sensitive topics such as drug use and sexual behaviors. However, the use of CASI for interviews should have minimized this problem [22, 59]. Finally, this kind of cross-sectional study does not allow us to evaluate the causality of the reported associations. Despite these limitations, our findings have important implications for understanding the risk factors of MA initiation, and the results will help in the development of new prevention or intervention programs for teenagers, adolescents, and adults in Myanmar.

## Conclusions

In conclusion, the current study provides an extensive description of gender similarities and differences in the age of initiation of MA use and factors affecting early initiation in Myanmar. The findings revealed that more than 60.0% of male and female users started using MA before the age of 18 years. Gender similarities and differences were also found across ethnic groups and with regard to reasons for first-time use of MA, places of first-time use, and risky sexual behaviors. Comprehensive and targeted MA prevention strategies and programs reflecting gender considerations are urgently needed in Myanmar. Moreover, gender-specific health education programs on MA misuse should ideally be implemented before the age of 14 years, which is the age when young people start to increasingly use MA. These programs should especially target ethnic minorities, employed MA users, and vulnerable populations such as bisexual/homosexual men, and those who exchange sex for money and/or drugs. Such programs should also consider entertainment venues as priority areas. Finally, such programs should also promote awareness and knowledge regarding MA and the effects thereof.

## Abbreviations

AOR: Adjusted odds ratio
ATS: Amphetamine-type stimulants
CASI: Computer assisted self-interviewing
CI: Confidence intervals
MA: Methamphetamine
RDS: Respondent driven sampling
SD: Standard deviation
STI: Sexually transmitted infection
